# Determinant components of newly onset versus improved metabolic syndrome in a population of Iran

**DOI:** 10.1038/s41598-020-76531-2

**Published:** 2020-11-11

**Authors:** Kamran Bagheri Lankarani, Behnam Honarvar, Parisa Keshani, Hadi Raeisi Shahraki

**Affiliations:** 1 Health Policy Research Center, Institute of Health, Shiraz University of Medical Sciences, Shiraz, Iran; 2 Shiraz HIV/AIDS Research Center, Institute of Health, Shiraz University of Medical Sciences, Shiraz, Iran; 3 Department of Biostatistics and Epidemiology, Faculty of Health, Shahrekord University of Medical Sciences, Shahrekord, Iran

**Keywords:** Endocrinology, Medical research, Risk factors

## Abstract

This study aimed to determine the risk factors related to regression and progression of metabolic syndrome, in a 4-year cohort study. A total of 540 individuals (≥ 18 years old) participated in both phase of the study. Participants were categorized into 3 categories of regressed, progressed and unchanged metabolic syndrome (MetS). Demographic, anthropometric and biochemical parameters were assessed for each individual in both phase. Variables differences (delta: Δ) between the two phase of study were calculated. Unchanged group was considered as baseline category. Based on IDF, MetS had been regressed and progressed in 42 participants (7.7%) and 112 (20.7%) participants respectively, in the second phase. More than 47% of people, whose MetS regressed, experienced also NAFLD regression. Results of multiple variable analysis revealed that increased age, positive Δ-TG, and Δ-FBS, significantly increased the odds of MetS progression based on IDF and ATP III definitions, while negative Δ-HDL and Δ-neutrophil to lymph ration increased the odds of progression. On the other hand, negative Δ-TG and positive Δ-HDL significantly increased the odds of Mets regression based of both IDF and ATP III. Management of hypertriglyceridemia, hyperglycemia, and HDL is a critical, non-invasive and accessible approach to change the trend of MetS.

## Introduction

Metabolic syndrome (MetS) as a non-communicable disease (NCD) had become epidemic in many countries of the world^1^. MetS is highly prevalent with multidimensional characteristics and a collection of abnormalities such as obesity, hypertension, dyslipidemia, and high blood glucose^2^.

Over the past 20 years, population of people with MetS, has significantly increased, which is directly associated to the global epidemic of obesity and diabetes. This is in addition to elevated threat of diabetes and cardiovascular disorders that arise from the MetS^3^ and influences more than 25% of the world population^4^.

The description by the Adult Treatment Panel III-2005 (ATP III) seems to be the most internationally accepted description. In 2009, the International Diabetes Federation (IDF) and the American Heart Association/National Heart, Lung and Blood Institute standardized their criteria for defining MetS^5^. The prevalence of MetS varies, based on the criteria used to define it. For instance, a study in Iran revealed the frequency of MetS to be 34.7% based on ATP III criteria, 37.4% based on IDF description, and 41.6% according to ATP III/AHA/NHLBI criteria^1^. In a systematic review and meta-analysis, according to ATP III criteria, MetS prevalence amongst people of Iran who were 20 years old or older was 23.8% and for under 20 years old was 10.9%^5^.

MetS risk factors were investigated in different studies. Central obesity was significantly associated with MetS mechanisms^6^, independent of insulin resistance^7^. Apart from the accessible intervention approaches, alterations such as suitable diet, regular exercise, anti-obesity medication and bariatric operation, and failure to manage MetS prevalence and its associated complications depend on the disease nature and patients not following the foregoing strategies^8^. Individuals with MetS are at higher risk of developing coronary artery disease (CAD). Coronary heart disease (CHD) were assessed by Framingham algorithms over a decade, and it was shown that many CHD incidences in MetS patients were prevented by controlling lipids and/or blood pressure^9^. This study is among scarce studies that assessed factors affect the progression and regression of the MetS have not been reported in any longitudinal study. Hence, this study aimed to investigate the risks related to regression and progression of MetS in a 4-year cohort study.

## Results

Total 540 participants completed both phase. Among all, 308 participants were female (56.9%). Mean for age and BMI were 43.4 ± 12.6 years, and 26.4 ± 4.3 kg/m^2^ respectively. Only 11.3% of the studied population had university education and 88% were married. Prevalence of metabolic syndrome based on IDF (and ATPIII) criteria in the first and second phase of the study, was 21.4% (22.2%) and 34.4% (36%), respectively. Based on IDF criteria, MetS had improved in 42 participants (7.7%) while 112 (20.7%) of the participants developed MetS after the second phase. These percentages were 42 (7.7%) and 117 (21.6%), respectively based on ATP III. Age, education, and employment status were significantly different between the three groups. Gender, marital status, physical activity and tobacco smoking and alcohol consumption were not significantly different between groups. All demographic correlates of MetS, based on IDF definition and ATP III are shown in Table1. Furthermore, para-clinical correlates of MetS was assessed based on the two criteria. Based on IDF, Δ-BMI, and Δ-WC, Δ-TG, Δ-FBS, Δ-SBP, Δ-DBP, Δ-lymphocyte and Δ-NLR were significantly different between the three groups of “Un-changed”, “Regressed” and “progressed”, while other lipid profile indices, HCT, kidney and liver function indices, neutrophil, platelet and daily energy intake were not different between groups, as Table 2 shows. History of diabetes, hyperlipidemia and hypertension or their treatments was different among groups. Among all who experienced MetS regression, NAFLD regressed in 47.6% and among participants, whose MetS progressed, NAFLD progression occurred in 18.7%.

**Table 1:** Baseline correlates of metabolic syndrome, based on IDF and ATP III criteria, in a population-based cohort study in Shiraz, Iran.

Variable	Metabolic syndrome (IDF definition)n (%)				Metabolic syndrome (ATP III definition)n (%)			
	Un-changed (n = 386)	Regressed (n = 42)	Progressed (n = 112)	Statistics (P-value)^**†**^	Un-changed (n = 381)	Regressed (n = 42)	Progressed (n = 117)	Statistics (P-value)^†^
**Age** (years, mean ± sd)	42.4 ± 12.6	47.7 ± 12.5	45.2 ± 11.5	0.007	42.1 ± 12.4	38.2 ± 13.3	45.8 ± 11.6	0.001
**Gender**				4.6 (0.10)				3.2 (0.2)
Male	179 (75.8)	13 (5.5)	44 (18.6)		173 (73.3)	13 (5.5)	50 (21.2)	
Female	208 (68.2)	29 (9.5)	68 (22.3)		209 (68.5)	29 (9.5)	67 (22.0)	
**Marital status**				2.4 (0.3)				2.0 (0.3)
Singles	51 (79.7)	4 (6.3)	9 (14.1)		50 (78.1)	4 (6.3)	10 (15.6)	
Married	336 (70.4)	38 (8.0)	103 (21.6)		332 (69.9)	38 (8.0)	107 (22.4)	
**Education**				8.6 (0.01)				8.0 (0.02)
≤ 12 years	224 (68.3)	34 (10.4)	70 (21.3)		223 (68.0)	34 (10.4)	71 (21.6)	
> 12yeras	163 (76.5)	8 (3.8)	42 (19.7)		159 (74.6)	8 (3.8)	46 (21.6)	
**Employment status**				7.2 (0.02)				6.0 (0.04)
Have job	216 (75.8)	15 (5.3)	54 (18.9)		211 (74.0)	15 (5.3)	59 (20.7)	
Have no job	171 (66.8)	27 (10.5)	58 (22.7)		171 (66.8)	27 (10.5)	58 (22.7)	
**Physical activity**^**‡**^				2.4 (0.3)				0.9 (0.6)
Yes	156 (75.4)	14 (6.8)	37 (17.9)		151 (72.9)	14 (6.8)	42 (20.3)	
No	231 (69.2)	28 (8.4)	75 (22.5)		231 (69.2)	28 (8.4)	75 (22.5)	
**Tobacco smoking**				0.01 (0.9)				0.04 (0.9)
Yes	74 (71.8)	8 (7.8)	21 (20.4)		72 (69.9)	8 (7.8)	23 (22.3)	
No	313 (71.5)	34 (7.8)	91 (20.8)		310 (70.8)	34 (7.8)	94 (21.5)	

*ATP III* Adult Treatment Panel III; IDF, International Diabetes Federation.

^**†**^Chi-squared test for qualitative variables, ANOVA test for quantitative.

**Table 2:** Baseline para-clinical correlates of metabolic syndrome, based on IDF and ATP III criteria in a population-based cohort study in Shiraz, Iran.

Variable	Metabolic syndrome (IDF definition)				Metabolic syndrome (ATP III definition)			
	Un-changed (n = 386)	Regressed (n = 42)	Progressed n = 112)	P-value	Un-changed (n = 381)	Regressed (n = 42)	Progressed (n = 117)	P-value
	(mean ± sd)	(mean ± sd)	(mean ± sd)		(mean ± sd)	(mean ± sd)	(mean ± sd)	
Δ-TG (mg/dl)	− 10.4 ± 69.1	− 62.6 ± 48.3	21.5 ± 77.2	< 0.001	− 12.1 ± 68.1	− 63.8 ± 47.1	25.8 ± 77.5	< 0.001
Δ-LDL (mg/dl)	− 11.9 ± 37.6	− 16.4 ± 38.4	− 5.7 ± 38.0	0.2	− 12.3 ± 37.7	− 16.4 ± 38.4	− 4.7 ± 37.5	0.1
Δ-HDL (mg/dl)	− 2.4 ± 16.9	1.7 ± 19.2	− 5.3 ± 17.4	0.07	− 2.2 ± 17.6	2.3 ± 18.5	− 6.0 ± 14.8	0.02
Δ-FBS (mg/dl)	1.4 ± 22.5	− 5.1 ± 46.7	10.2 ± 34.9	0.003	1.3 ± 22.3	− 6.8 ± 47.3	11.0 ± 34.4	< 0.001
Δ-BUN (mg/dl)	− 0.9 ± 5.4	− 0.6 ± 3.4	− 1.9 ± 10.4	0.3	− 1.0 ± 5.4	− 0.3 ± 3.5	− 1.8 ± 10.2	0.4
Δ-SBP (mm Hg)	− 0.3 ± 14.9	− 2.8 ± 15.1	6.4 ± 15.9	< 0.001	− 0.4 ± 14.9	− 3.4 ± 14.8	6.1 ± 15.6	< 0.001
Δ-DBP (mm Hg)	1.6 ± 10.7	− 1.8 ± 8.8	6.4 ± 12.0	< 0.001	1.6 ± 10.8	− 2.2 ± 8.4	6.2 ± 11.8	< 0.001
Δ-Cr (mg/dl)	− 0.1 ± 0.3	− 0.1 ± 0.2	− 0.1 ± 0.3	0.9	− 0.1 ± 0.3	− 0.1 ± 0.2	− 0.1 ± 0.3	0.9
Δ-AST (U/L)	− 4.3 ± 16.3	− 7.6 ± 13.2	− 4.1 ± 10.6	0.3	− 4.3 ± 16.4	− 7.6 ± 13.2	− 4.0 ± 10.6	0.3
Δ-ALT (U/L)	− 4.8 ± 18.7	− 10.1 ± 20.6	− 3.1 ± 14.8	0.1	− 4.9 ± 18.8	− 10.3 ± 20.5	− 2.8 ± 14.6	0.07
Δ-AST/ALT	0.01 ± 0.4	0.02 ± 0.3	0.04 ± 0.4	0.8	0.01 ± 0.4	0.03 ± 0.3	0.02 ± 0.4	0.9
Δ-HCT (%)	− 0.2 ± 4.1	− 1.6 ± 6.2	− 0.6 ± 2.7	0.07	− 0.1 ± 4.1	− 1.6 ± 6.2	− 0.7 ± 2.7	0.07
Δ-Neutrophil (%)	− 1.1 ± 11.3	− 1.5 ± 10.0	− 3.1 ± 12.0	0.2	− 1.0 ± 11.3	− 1.9 ± 10.4	− 3.1 ± 11.8	0.2
Δ-Lymph (%)	2.9 ± 9.2	2.0 ± 8.6	5.3 ± 7.7	0.03	2.9 ± 9.2	2.1 ± 8.6	5.2 ± 7.6	0.03
Δ-Platelet × 10^3^ (count/μl)	− 16.1 ± 48.6	− 12.6 ± 72.2	− 14.7 ± 54.6	0.9	− 16.9 ± 48.8	− 11.4 ± 72.3	− 12.5 ± 53.8	0.6
Δ-NLR	− 0.2 ± 0.9	− 0.2 ± 1.2	− 0.5 ± 1.3	0.04	− 0.2 ± 0.9	− 0.2 ± 1.2	− 0.5 ± 1.3	0.04
Δ-BMI (kg/m^2^)	0.1 ± 2.5	− 0.9 ± 1.9	0.7 ± 2.4	< 0.001	0.1 ± 2.2	− 0.9 ± 1.9	0.7 ± 2.3	< 0.001
Δ-WC (cm)	7.6 ± 8.4	5.0 ± 6.0	10.5 ± 8.0	< 0.001	7.6 ± 8.4	5.3 ± 5.8	10.0 ± 8.1	0.002
Δ-Kcal (daily intake)	226.2 ± 666.2	247.9 ± 502.7	240.4 ± 791.4	0.9	229.4 ± 662.5	245.1 ± 504	230.3 ± 796.4	0.9
**NAFLD n (%)**								
Un-changed	209 (54.1)	19 (45.2)	61 (54.4)	0.002	207 (54.3)	19 (45.2)	63 (53.8)	0.01
Regressed	110 (28.4)	20 (47.6)	30 (26.7)		107 (28.0)	20 (47.6)	33 (28.2)	
Progressed	67 (17.3)	3 (7.1)	21 (18.7)		67 (17.5)	3 (7.1)	21 (17.9)	

*ALT* alanine aminotransferase; *AST* aspartate aminotransferase, *ATP III* adult treatment panel III; *BMI* body mass index; *BUN* blood urea nitrogen; *Cr* creatinine; *DBP* diastolic blood pressure; *FBS* fasting blood sugar; *Hb* hemoglobin; *HCT* hematocrit; *HDL* high density lipoprotein-cholesterol; *IDF* International Diabetes Federation; *Kcal* kilocalorie; *LDL* low density lipoprotein-cholesterol, *NAFLD* non-alcoholic fatty liver disease; *NLR* neutrophil to lymph ratio; *SBP* systolic blood pressure; *TG* triglyceride; *WC* waist circumference.

^†^ANOVA test.

**Figure 1:**
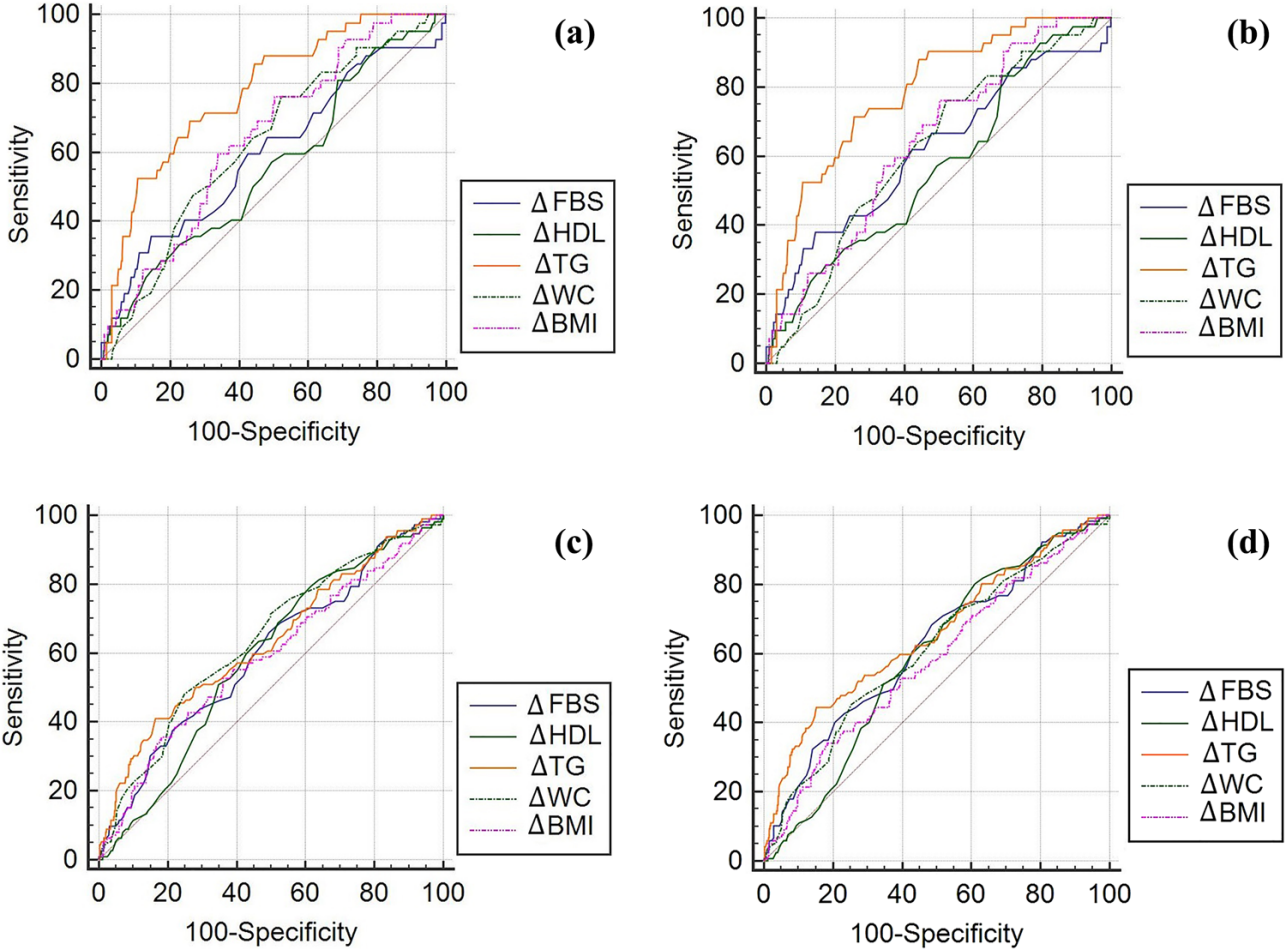
Cut points and area under ROC Curve (AUC) for para-clinical variables affected. (**a**) Regressed metabolic syndrome groups, based on IDF criteria. (**b**) Regressed metabolic syndrome groups, based on ATP III criteria. (**c**) Progressed metabolic syndrome groups, based on IDF criteria. (**d**) Progressed metabolic syndrome groups, based on ATP III criteria.

Area under the curves (AUC) for differentiating regressed or progressed cases versus others was obtained for significant variables extracted from Table 2. Δ-TG was the only variable with AUC greater than 0.70 for differentiating regressed cases and based on IDF, with a decrease of at least 40 mg/dl, could predict the disease's regression by 78%. BMI alteration at least 0.28 unit can help to MetS regression, while alteration more than 1.62 (kg/m^2^) can predict disease progression. Decreasing WC (at least 8 cm), diastolic blood pressure (at least 9 mmHg), and FBS (at least 14 mg/dl), could predict MetS regression by 63%, 0.61% and 0.60% respectively, based on IDF criteria (Fig. 1, Table 3).

**Table 3:** Cut points and area under ROC curve (AUC) for para-clinical variables associated with regression and progression of metabolic syndrome.

Variables	IDF				ATP III			
	Regressed^a^		Progressed^b^		Regressed^a^		Progressed^b^	
	Cut point	AUC	Cut point	AUC	Cut point	AUC	Cut point	AUC
Δ-TG (mg/dl)	$40$	0.78	38	0.63	$40$	0.79	38	0.66
Δ-BMI (kg/m^2^)	0.28	0.65	1.62	0.59	0.28	0.64	1.62	0.58
Δ-WC (cm)	$8$	0.63	11	0.64	$8$	0.62	11	0.61
Δ-DBP (mm Hg)	$9$	0.61	5.5	0.61	$9$	0.63	10	0.61
Δ-FBS (mg/dl)	$14$	0.60	3	0.60	$14$	0.62	3	0.62
Δ-SBP (mm Hg)	$20$	0.56	5.5	0.63	$3$	0.58	5.5	0.63
Δ-HDL (mg/dl)	11	0.56	$2$	0.59	11	0.57	$2$	0.59

*ATP III* adult treatment panel III; *AUC* area under the ROC curve; *BMI* body mass index; *DBP* diastolic blood pressure; *FBS* fasting blood sugar; *HDL* high density lipoprotein-cholesterol; *IDF* International Diabetes Federation; *SBP* systolic blood pressure; *TG* triglyceride; *WC* waist circumference.

^a^Regressed versus progressed and unchanged.

^b^Progressed versus regressed and unchanged.

As Table 4 shows the finding of multiple variable analysis, increased Δ-TG (OR 1.01; P < 0.001 and OR 1.01; P < 0.001) and Δ-FBS (OR 1.01; P = 0.02 and OR 1.01; P = 0.01) and declining Δ-NLR (OR 0.81; P = 0.04 and OR 0.79, P = 0.02), and Δ-HDL (OR 0.98; P = 0.05 and OR 0.97; P = 0.001) significantly increased the odds of MetS progression based on both IDF and ATP III definitions, respectively.

**Table 4:** Multiple variable analysis of correlates of metabolic syndrome, based on new IDF and ATP III criteria in a population-based cohort study in Shiraz, Iran.

	Progressed (IDF definition)		Progressed (ATP3 definition)		Regressed (IDF definition)		Regressed (ATP3 definition)	
	OR (CI 95%)	P-value	OR (CI 95%)	P-value	OR (CI 95%)	P-value	OR (CI 95%)	P-value
Age (year)	1.0 (1.0, 1.0)	0.1	1.0 (1.0, 1.0)	0.004	1.0 (0.9, 1.0)	0.3	1.0 (0.9, 1.0)	0.1
Δ-BMI (kg/m^2^)	1.2 (0.9, 1.5)	0.1	1.2 (0.9, 1.5)	0.09	0.9 (0.6, 1.3)	0.7	0.9 (0.6, 1.3)	0.7
Δ-WC (cm)	1.2 (0.9, 1.5)	0.1	1.1 (0.8, 1.5)	0.3	1.2 (0.8, 1.9)	0.2	1.2 (0.8, 1.8)	0.3
Δ-TG (mg/dl)	1.0 (1.0, 1.0)	< 0.001	1.0 (1.0, 1.0)	< 0.001	0.9 (0.9, 0.9)	< 0.001	0.9 (0.9, 0.9)	< 0.001
Δ-HDL (mg/dl)	0.9 (0.9, 0.9)	0.05	0.9 (0.9, 0.9)	0.001	1.0 (1.0, 1.0)	0.003	1.0 (1.0, 1.0)	0.001
Δ-FBS (mg/dl)	1.0 (1.0, 1.0)	0.02	1.0 (1.0, 1.0)	0.01	1.0 (0.9, 1.0)	0.8	1.0 (0.9, 1.0)	0.5
Δ-Hb (g/dl)	0.7 (0.5, 1.0)	0.1	0.7 (0.5, 1.0)	0.08	0.8 (0.5, 1.1)	0.2	0.7 (0.4, 1.1)	0.1
Δ-HCT (%)	1.0 (0.9, 1.1)	0.7	1.0 (0.9, 1.1)	0.7	0.9 (0.8, 1.0)	0.6	1.0 (0.8, 1.1)	0.9
Δ-NLR	0.8 (0.6, 0.9)	0.04	0.7 (0.6, 0.9)	0.02	0.9 (0.6, 1.2)	0.6	0.9 (0.6, 1.2)	0.5
Δ-ALT (U/L)	1.0 (0.9, 1.0)	0.5	1.0 (0.9, 1.0)	0.4	0.9 (0.9, 1.0)	0.1	0.9 (0.9, 1.0)	0.5
Δ-SBP (mm Hg)	1.0 (1.0, 1.0)	0.1	1.0 (0.9, 1.0)	0.1	0.9 (0.9, 1.0)	0.7	0.9 (0.9, 1.0)	0.09
Δ-DBP (mm Hg)	1.0 (1.0, 1.0)	0.1	1.0 (1.0, 1.0)	0.09	0.9 (0.9, 1.0)	0.1	0.9 (0.9, 1.0)	0.1
Gender: male/female (as reference)	1.0 (0.5, 2.0)	0.9	1.2 (0.6, 2.5)	0.5	1.0 (0.3, 3.1)	0.9	0.9 (0.3, 3.1)	0.9
Education: > 12 years/ ≤ 12 years (as reference)	1.1 (0.6, 1.9)	0.7	0.9 (0.5, 1.4)	0.6	0.5 (0.2, 1.4)	0.2	0.4 (0.1, 1.2)	0.1
Have job/Have no job (as reference)	0.7 (0.3, 1.4)	0.5	0.6 (0.3,1.3)	0.2	0.5 (0.1, 1.5)	0.2	0.4 (0.1,1.4)	0.2

*ALT* alanine aminotransferase; *ATP III* adult treatment panel III; *BMI* body mass index; *DBP* diastolic blood pressure; *FBS* fasting blood sugar; *Hb* hemoglobin; *HCT* hematocrit; *HDL* high density lipoprotein-cholesterol; *IDF* International Diabetes Federation; *NLR* neutrophil to lymph ratio; *SBP* systolic blood pressure; *TG* triglyceride; *WC* waist circumference.

Declining Δ-TG (OR 0.99; p < 0.001 and OR 0.99; p < 0.001), and increasing Δ-HDL (OR 1.03; p = 0.003 and OR 1.03; p = 0.001), significantly increased the odds of MetS regression based on both IDF and ATP III. Other variables did not affect the disease regression and progression, significantly.

## Discussion

In this cohort study, association between demographic, social, anthropometric and dietary risk factors with the MetS regression or progression were assessed. Based on the univariable analysis, variables of age, Δ-BMI, Δ-WC, Δ-FBS, Δ-TG, Δ-Hb, Δ-lymph and Δ-NLR, Δ-SBP, employment status and education were associated with changing of the disease status while Δ-TG, Δ-FBS, Δ-HDL and Δ-NLR were effective in regression and progression of the disease using multiple variable analysis.

Using ATP III as a description of MetS, five more participants were placed in the progressed group in comparison to IDF, with no difference to regressed group. ATP III was the most agreed criteria for MetS and the most widely used^10^. Amongst the Iranian population, IDF definition for MetS is in good correlation with ATP III definition, but in low correlation with the WHO definition^11^. In the Middle Eastern population, ATP III criteria can better predict high CVD risk scores, and IDF criteria is a better predictor of pre-diabetes and diabetes, while IDF and ATP III definition provides similar frequency rate of MetS^12^. MetS was higher amongst non-diabetics who had impaired glucose, using ATP III criteria compare to IDF^12^. The two definitions were overlapped for 93% of the individuals in defining the occurrence or nonexistence of the MetS in the National Health and Nutrition Examination Survey (NHANES)^13^. When these classifications were applied to an urban population in the United States, the IDF criteria categorized 15–20% more adults with MetS than the ATP III criteria^14^.

### Demographic and social risk factors related to metabolic syndrome regression or progression

Although gender was not a significant risk factor in our study, there was a significant age factor between the three groups. We found that the occurrence of MetS has an association with age. In Marquezine et al. study^15^, general prevalence of MetS was not significantly different between genders, but a clear relation was established between the prevalence and progressing of age. In another study, the prevalence of MetS significantly increased with age only among male participants^16^, while in other study it increased in both men and women^17^.

In our study, employment and education level as an indication of social status was linked to the MetS in the univariable analysis, and similar results were reported in some other studies. Lower social economic status was significantly associated with the risk of MetS in women, but not in men^15^. The prevalence of MetS was inversely associated with education level between women^17^.

The occurrence of MetS had significantly increased amongst female blue-collar workers, those with lower education level and household income^16^. Part-time or temporary workers of either gender showed higher MetS prevalence than full-time workers^16^, but job rank was not associated with MetS in Mehrdad et al. study^18^. The prevalence of MetS significantly augmented with being married^16^, and smoking status^16,19^. The odds ratios (ORs) of suffering from MetS was significantly higher amongst people who smoked at least 20 cigarettes/day^20^. However, in our study, smoking states was not significantly different between the groups.

### Anthropometric and life style risk factors correlated to the metabolic syndrome regression or progression

Δ-WC and Δ-BMI in univariable analysis were significantly different amongst the groups. Additionally, Δ-WC increased the odds of disease progression. Abdominal obesity is an indicator of body fat which is closely associated with MetS. Therefore, a significant reduction in weight is able to decrease all risk factors related to MetS, also reducing the risk of type 2 diabetes^21^.

Studies revealed that just calorie balance could not solve clinical problems^22^. Decreasing dietary carbohydrate proposed to be an effective way to improve MetS^23,24^ and western dietary pattern, increased the occurrence of MetS^25^. Dietary factors such as energy, carbohydrate, and fat and protein intake were not associated with altering MetS status in our population. Additionally, in our study physical activity was not correlated to progression and regression of the disease. Although other studies have emphasized on physical activity effect on MetS, its prevalence was negatively associated with the level of physical activity^17,20^. Different guidelines are currently proposing regular, practical, and moderate regimens of physical activity such as 30 min daily moderate-intensity exercise. Continuous and regular physical activity is able to decrease all the risk factors associated with MetS^3^. Lifestyle adjustment and weight loss should be the main principal while treating or avoiding MetS and its mechanisms^26^.

### Para-clinical parameters associated with metabolic syndrome regression or progression

In our study, through a univariable analysis, Δ-FBS, Δ-TG, Δ-HDL, Δ-lymph, Δ-NLR, Δ-SBP and Δ-DBP were among the variables correlated with the MetS. Furthermore, in multivariable analysis Δ-FBS, Δ-TG, Δ-HDL were significantly associated with the MetS.

Δ-FBS was different between regressed and progressed groups. Since impaired fasting glucose is a component of ATP-III and IDF definitions, this finding might not be surprising^3^. While MetS and type 2 diabetes (T2D) often co-occur, those individuals with MetS without diabetes are at higher risk of developing diabetes^2^. Several studies have indicated that MetS is a predictor of future diabetes^3^.

There was a positive and significant association between MetS diagnosis and some MetS components such as TG, HDL and LDL in some studies (gentile, 2008). Comprised of elevated serum TG, small LDL particles, and HDL cholesterol, the lipid triad is a constituent of MetS associated with CAD^27^. Research has shown that hypertriglyceridemia has a strong relationship with atherogenic factors^27^. And evidence is stronger for triglyceride as a synergistic CAD risk factor among other dyslipidemias^28^. Framingham algorithms have shown that many CHD events in patients with MetS might be preventable through the control of lipids^9^. LDL addressed as a valuable marker for diagnosis and severity of the MetS in some studies^29^. However, one of the main disruption in MetS is the reduction of HDL, which is due to changes in HDL composition and metabolism^3^. HDL increase to normal levels prevented 25.3 and 27.3% of CHD in men and women, respectively, and the optimal control precluded 51.2 and 50.6% of the incidences^9^. Overall it seems that through the controlling of lipid profile, it would be possible to manage CHD in patients with MetS.

Reducing the blood pressure down to normal levels, prevented 28.1 and 12.5% of CHD events in MetS men and women, respectively. And, controlling it at optimal levels led to preventing CHD to 28.2% and 45.2% of events, respectively^9^.

Δ-HCT was not different between groups, but Δ-Hb was different between regressed, progressed and un-changed groups in a univariable analysis. In similar studies, participants with MetS had elevated hemoglobin and ferritin concentration^30,31^. Higher Hb levels were related to all MetS mechanisms^30^. In a large cohort study, there was no association between Hb concentration and the incidence of MetS amongst women; however, it was a risk factor for the incidence of MetS in men^32^.

From the aspects of immunity, lymph and NLR were significantly different in regressed, progressed and un-changed groups in our study. We found that decreasing NLR was associated with the disease progression, which was not congruent with previous studies. In one systematic review and meta-analysis, including thirty-eight article, high NLR was significantly associated with the risks of coronary artery disease, acute coronary syndrome, stroke and combined cardiovascular events^33^; however, at the initial stage of the disease, NLR did not correlated with the cardiovascular determinants^34^. In Nordestgaard et al.^35^ study neutrophil and NLR were not significant determinants for Myocardial Infarction in the seemingly healthy population. Furthermore, in a 9-year cohort study, NLR was not a predictor of hypertension in participants lower than 60 years old, female and BMI-specific groups^36^.

NAFLD is known as the hepatic expression and a strong determinant of the MetS^37,38^. Glucose and triglycerides as the two main components of MetS are produced more by the fatty liver^39^ and lead to the development of the MetS, which has potentially related to clinical consequences for preventing and handling MetS^38^. Around 90% of the subjects with NAFLD had ≥ 1 features of metabolic syndrome, and approximately 33% had the complete diagnosis^40^. In our study more than 47% of people, whose MetS regressed, experienced also NAFLD regression. Furthermore, both MetS and NAFLD progressed in 18.7% of participants during the study. According to the association between NAFLD and MetS, and high prevalence of NAFLD in Iran^41^, considering the factors related to NAFLD regression would be valuable to lead MetS regression.

We didn’t assessed the rural that consist about 20% of the province, due to possible difference in their life style with urban population. We tried also to use our regional cut point for WC, but due to heterogeneity to report about this parameter^42,43^, we prefer to use European cut point which is suggested for Eastern Mediterranean and Middle East populations^44^. Furthermore, some factors such as genetics, and income could not be evaluated easily due to high budget demanding and no appropriate tool respectively. Circadian rhythm was not measured in this study as well, should be considered in future studies. Although the sample size was not so high because of the nature of the study and the logistic challenges but it was tried to conduct the study with the maximum possible logistic and budget capacity while an attempt was made to have a representative sample size in the community population by using accurate randomization to introduce different socio-economic classes of the community in the study.

## Conclusion

After adjusting for confounders, increased TG, FBS and decreased NLR and HDL significantly increased the odds of metabolic syndrome progression. Furthermore, declining TG and increasing HDL significantly increased the odds of metabolic syndrome regression based on IDF and ATP III definitions. As a practical note, TG and HDL can be considered as the most important determinant of metabolic syndrome change. Management of hypertriglyceridemia, hyperglycemia, and HDL is a critical, non-invasive and accessible approach to change the trend of MetS.

## Materials and methods

The present cohort study was conducted in Shiraz, a 2 million populated city in southern Iran, in two phase. A multistage cluster random sampling, based on postal codes was performed. Participants were randomly selected from residents aged 18 years or older in all the seven postal code districts of Shiraz. These districts consisted of different socio-economic groups that more or less are similar to the urban population in the megacities of Iran.

The second phase of this study was carried out and lasted up to 2018, and all the first phase steps were repeated. Two physicians and two nurses conducted the interviews, achieved the medical histories and did the physical tests. Physicians were gender identical for all participants. Among 819 people who participate the first phase, 540 completed phase 2 study. Regarding comparison between who participated and who did not participate in the second phase of this study, the prevalence of MetS and its components was not significantly different between them, except for blood pressure and FBS, which were lower in the dropped out ones.

According to the ATP III and IDF criteria, for diagnose of MetS criteria in the two phase, the participants were categorized into three groups: (1) Un-change group (have no MetS at both phase or no change in their MetS status) (2) regressed group, which included those who were suffering from MetS in phase 1, but their status had improved with no evident MetS in the second phase, (3) progressed group, included participants who had no MetS in the first phase but were afflicted with the disease in the second phase. Fourteen participants were known case of MetS at first phase also remain in that status in second phase, and we compare statistical parameter of this group with healthy group and as we didn’t see any significant difference between them, they were added to un-changed group for analysis.

New IDF definition for MetS defines it as having: Central obesity (defined as waist circumference (WC) ≥ 91.5 cm for men and ≥ 85.5 cm for women, based on Iranian WC cutoff point for diagnosing^42^). Raised TG level: ≥ 150 mg/dL (1.7 mmol/L), or specific treatment for this lipid abnormality. Reduced HDL: < 40 mg/dL (1.03 mmol/L) in males and < 50 mg/dL (1.29 mmol/L) in females, or specific treatment for this lipid abnormality. Raised blood pressure: systolic BP ≥ 130 or diastolic BP ≥ 85 mm Hg, or treatment of previously diagnosed hypertension. Raised fasting plasma glucose (FPG) ≥ 100 mg/dL (5.6 mmol/L), or previously diagnosed type 2 diabetes^44^.

ATP III for MetS defines as three or more of the following five risk factors: Waist circumference in Men > 102 cm (> 40 in), in women > 88 cm (> 35 in), Triglycerides ≥ 150 mg/dL (1.7 mmol/L), HDL in Men < 40 mg/dL (1.03 mmol/L), in Women < 50 mg/dL (1.29 mmol/L), Blood pressure ≥ 130/ 85 mm Hg, Fasting glucose ≥ 110 mg/dL (6.1 mmol/L)^45^.

### Measurements

At the baseline, a checklist including demographic, social characteristics and medical history (includes diseases and medications) were completed via face to face interview. Information such as participant’s marital status (single/married), education (less/ higher than 12 years), job status (Have job/Have no job) and tobacco smoking, alcohol consumption were collected at the baseline. Dietary intake was measured in both phase of the study, using a validated, Persian version, food frequency questionnaire (FFQ)^46^ which were asked from participants face to face, filled by trained interviewers and analyzed via Nutritionist-4 software modified for Persian food. Participant who has activity for a weekly minimum of 150–300 min of moderate-intensity, or 75–150 150 min of vigorous-intensity, or a mixture between the two phases, considered as physically active person^47^. Physical activity was self-reported by interviewees.

Height was measured by the use of a tape measure to the nearest of 0.1 cm and weight was measured to the nearest of 0.1 kg, wearing light clothes. Body Mass Index (BMI) was calculated as weight/height^2^ (kg/m^2^). Waist circumference was measured as the distance around the waist between the lowest rib and iliac crest and above the umbilicus using a non-stretchable tape measure. Diagnosis of non-alcoholic fatty liver (NAFLD) was performed via upper abdominal ultrasonography (US) according to the augmented hepatic parenchyma echogenicity with the attenuation in the portal vein or diaphragm echogenicity. Using a Shimadzu ultrasound machine (Shimadzu Inc., Tokyo, Japan) with a 5-MHz to 7-MHz transducer probe (curvilinear), the trans-abdominal ultrasonography was conducted. An expert radiologist performed all the ultrasonogrphic evaluations.

After 8-h fasting, 3-mL blood sample was taken and centrifuged within 30 min after collection and stored at − 20 °C until further analysis. Fasting blood sugar (FBS), blood urea nitrogen (BUN), creatinine (Cr), alanine aminotransferase (ALT) and aspartate aminotransferase (AST), lipid profile consists of triglyceride (TG), low (LDL) and high (HDL) density lipoprotein, total cholesterol (Chol), cell blood count (CBC) including hemoglobin (Hb), hematocrit (HCT), neutrophils, lymphocyte, neutrophil to Lymph ratio (NLR), and platelet were measured. Systolic blood pressure (SBP) and diastolic blood pressure (DBP) were measured according to the world health organization (WHO) criteria, for each individual in both phase, in sitting and supine position in two 15 min apart. The laboratory technician, and laboratory analysis conditions were similar in both phase of the study to reduce any bias.

### Ethical standards disclosure

This study was conducted according to the guidelines laid down in the Declaration of Helsinki and all procedures involving research study participants were approved by the Shiraz University of Medical Sciences (SUMS) ethics board committee, reference number: IR.SUMS.REC.1397.312. Written informed consent was obtained from all participants and questionnaires were anonymous and encoded. For the participants, all processes were free of charge, and interviews were conducted individually by the same gender. Participants with diagnosed non-alcoholic fatty liver were referred to a specialist.

### Statistical analysis

Analysis was performed by IBM SPSS statistical software version 24. Data are expressed as median and frequency percentage. For quantitative variables, differences (phase 2-phase 1, delta: Δ) between the two phase of study were calculated. Analysis of variance (ANOVA) and Chi-squared tests were used as univariable analysis. In the next step, variables with P value less than 0.2 in univariable analysis, were entered into the multi-nominal logistic regression analysis to examine the odds of regression and progression of MetS, while un-changed group was considered as baseline category. Receiver Operating Characteristic (ROC) analysis was done for significant variable (p < 0.10). Collinearity among variables was assessed using variance inflation factor (VIF) before multivariate analysis and no factors were correlated (VIF < 5). P value less than 0.05 was considered significant in all final analysis.

## References

[1] Saklayen MG (2018). The global epidemic of the metabolic syndrome. Curr. Hypertens. Rep..

[2] Punthakee Z, Goldenberg R, Katz P (2018). Definition, classification and diagnosis of diabetes, prediabetes and metabolic syndrome. Can. J. Diabetes.

[3] Eckel RH, Grundy SM, Zimmet PZ (2005). The metabolic syndrome. The Lancet..

[4] Ľudmila P, František B, Paralič J (2018). Data analytics for metabolic syndrome diagnostics. World Congress Med. Phys. Biomed. Eng..

[5] Mazloomzadeh S, Rashidi Khazaghi Z (2018). The prevalence of metabolic syndrome in Iran: a systematic review and meta-analysis. Iran J Public Health..

[6] Fezeu L, Balkau B, Kengne A-P, Sobngwi E, Mbanya J-C (2007). Metabolic syndrome in a sub-Saharan African setting: central obesity may be the key determinant. Atherosclerosis.

[7] Onat A (2007). Determinants and definition of abdominal obesity as related to risk of diabetes, metabolic syndrome and coronary disease in Turkish men: a prospective cohort study. Atherosclerosis.

[8] Vazzana N, Santilli F, Sestili S, Cuccurullo C (2011). Determinants of increased cardiovascular disease in obesity and metabolic syndrome. Curr. Med. Chem..

[9] Wong ND, Pio JR, Franklin SS, Gil JL, Kamath TV, Williams GR (2003). Preventing coronary events by optimal control of blood pressure and lipids in patients with the metabolic syndrome. Am. J. Cardiol..

[10] Alberti KG (2009). Harmonizing the metabolic syndrome: a joint interim statement of the International Diabetes Federation Task Force on Epidemiology and Prevention; National Heart, Lung, and Blood Institute; American Heart Association; World Heart Federation; International Atherosclerosis Society; and International Association for the Study of Obesity. Circulation.

[11] Zabetian A, Hadaegh F, Azizi F (2007). Prevalence of metabolic syndrome in Iranian adult population, concordance between the IDF with the ATPIII and the WHO definitions. Diabetes Res. Clin. Pract..

[12] Hajat C, Shather Z (2012). Prevalence of metabolic syndrome and prediction of diabetes using IDF versus ATPIII criteria in a Middle East population. Diabetes Res. Clin. Pract..

[13] Ford ES (2005). Prevalence of the metabolic syndrome defined by the International Diabetes Federation among adults in the U.S. Diabetes Care.

[14] Adams RJ (2005). Population comparison of two clinical approaches to the metabolic syndrome implications of the new International Diabetes Federation consensus definition. Diabetes Care.

[15] Marquezine GF, Oliveira CM, Pereira AC, Krieger JE, Mill JG (2008). Metabolic syndrome determinants in an urban population from Brazil: social class and gender-specific interaction. Int. J. Cardiol..

[16] Cho D, Koo J-W (2018). Differences in metabolic syndrome prevalence by employment type and sex. Int. J. Environ. Res. Public Health..

[17] Li Y, Zhao L, Yu D, Wang Z, Ding G (2018). Metabolic syndrome prevalence and its risk factors among adults in China: a nationally representative cross-sectional study. PLoS ONE.

[18] Mehrdad R, Pouryaghoub G, Moradi M (2018). Association between metabolic syndrome and job rank. Int. J. Occup. Environ. Med..

[19] Cai H (2012). Prevalence and determinants of metabolic syndrome among women in Chinese rural areas. PLoS ONE.

[20] Xu X (2018). The influence of diet and behaviour on metabolic syndrome and the prevalence of metabolic syndrome according to different definitions in west China. Asia Pac. J. Clin. Nutr..

[21] Aganović I, Dušek T (2007). Approach to the treatement of metabolic syndrome. EJIFCC.

[22] Bando H (2018). Calorie balance model and carbohydrate-insulin model. J. Obes. Treat. Weight Manag..

[23] Kwon Y-J, Lee H-S, Lee J-W (2018). Association of carbohydrate and fat intake with metabolic syndrome. Clin. Nutr..

[24] Volek JS (2008). Carbohydrate restriction has a more favorable impact on the metabolic syndrome than a low fat diet. Lipids.

[25] Lutsey PL, Steffen LM, Stevens J (2008). Dietary intake and the development of the metabolic syndrome, the atherosclerosis risk in communities study. Circulation.

[26] Cornier M-A (2008). The metabolic syndrome. Endocr. Rev..

[27] Grundy SM (1998). Hypertriglyceridemia, atherogenic dyslipidemia, and the metabolic syndrome. Am. J. Cardiol..

[28] Gotto AM (1998). Triglyceride as a risk factor for coronary artery disease. Am. J. Cardiol..

[29] Gentile M (2008). Small dense LDL particles and metabolic syndrome in a sample of middle-aged women. Findings from Progetto Atena. Clin. Chim. Acta..

[30] Hämäläinen P, Saltevo J, Kautiainen H, Mäntyselkä P, Vanhala M (2012). Erythropoietin, ferritin, haptoglobin, hemoglobin and transferrin receptor in metabolic syndrome: a case control study. Cardiovasc. Diabetol..

[31] Laudisio A, Bandinelli S, Gemma A, Ferrucci L, Antonelli IR (2013). Metabolic syndrome and hemoglobin levels in elderly adults: the Invecchiare in Chianti Study. J. Am. Geriatr. Soc..

[32] Hashimoto Y (2015). Hemoglobin concentration and incident metabolic syndrome: a population-based large-scale cohort study. Endocrine.

[33] Angkananard T, Anothaisintawee T, McEvoy M, Attia J, Thakkinstian A (2018). Neutrophil lymphocyte ratio and cardiovascular disease risk: a systematic review and meta-analysis. BioMed Res. Int..

[34] Caimi G, Lo Presti R, Canino B, Ferrera E (2016). Behaviour of the neutrophil to lymphocyte ratio in young subjects with acute myocardial infarction. Clin. Hemorheol. Microcirc..

[35] Nordestgaard BG, Adourian AS, Freiberg JJ, Guo Y, Muntendam P, Falk E (2010). Risk factors for near-term myocardial infarction in apparently healthy men and women. Clin. Chem..

[36] Jhuang Y-H (2019). Neutrophil to lymphocyte ratio as predictor for incident hypertension: a 9-year cohort study in Taiwan. Hypertens. Res..

[37] Vannia E (2010). From the metabolic syndrome to NAFLD or vice versa?. Dig. Liver Dis..

[38] Lonardo A, Ballestri S, Marchesini G, Angulo P, Loria P (2015). Nonalcoholic fatty liver disease: a precursor of the metabolic syndrome. Dig. Liver Dis..

[39] Yki-Järvinen H (2014). Non-alcoholic fatty liver disease as a cause and a consequence of metabolic syndrome. Lancet Diabetes Endocrinol..

[40] Marchesini G (2003). Nonalcoholic fatty liver, steatohepatitis, and the metabolic syndrome. Hepatology.

[41] Honarvar B, Bagheri Lankarani K, Keshani P, Rafiee T (2017). Dietary determinants of non-alcoholic fatty liver disease in lean and non-lean adult patients: a population-based study in Shiraz, Southern Iran. Hepat. Mon..

[42] Esteghamati A, Ashraf H, Rashidi A, Meysamie A (2008). Waist circumference cut-off points for the diagnosis of metabolic syndrome in Iranian adults. Diabetes Res. Clin. Pract..

[43] Azizi F (2010). Appropriate waist circumference cut-off points among Iranian adults: the first report of the Iranian National Committee of Obesity. Arch. Iran. Med..

[44] Federation. ID. *Metabolic syndrome-The IDF consensus worldwide definition of the METABOLIC SYNDROME*. Brussels, Belgium: IDF Communications. https://www.idf.org/component/attachments/attachments.html?id=705&task=download. (2006)

[45] Huang PL (2009). A comprehensive definition for metabolic syndrome. Dis. Models Mech..

[46] Mirmiran P, Esfahani FH, Mehrabi Y, Hedayati M, Azizi F (2010). Reliability and relative validity of an FFQ for nutrients in the Tehran Lipid and Glucose Study. Public Health Nutr..

[47] Piercy KL (2018). The physical activity guidelines for Americans. JAMA.

